# Identifying conservation technology needs, barriers, and opportunities

**DOI:** 10.1038/s41598-022-08330-w

**Published:** 2022-03-21

**Authors:** Nathan R. Hahn, Sara P. Bombaci, George Wittemyer

**Affiliations:** 1 Department of Fish, Wildlife, and Conservation Biology, Colorado State University, 1474 Campus Delivery, Fort Collins, CO 80523 USA; 2 Graduate Degree Program in Ecology, Colorado State University, Fort Collins, CO USA

**Keywords:** Ecology, Biodiversity, Conservation biology

## Abstract

Amid accelerating threats to species and ecosystems, technology advancements to monitor, protect, and conserve biodiversity have taken on increased importance. While most innovations stem from adaptation of off-the-shelf devices, these tools can fail to meet the specialized needs of conservation and research or lack the support to scale beyond a single site. Despite calls from the conservation community for its importance, a shift to bottom-up innovation driven by conservation professionals remains limited. We surveyed practitioners, academic researchers, and technologists to understand the factors contributing to or inhibiting engagement in the collaborative process of technology development and adoption for field use and identify emerging technology needs. High cost was the main barrier to technology use across occupations, while development of new technologies faced barriers of cost and partner communication. Automated processing of data streams was the largest emerging need, and respondents focused mainly on applications for individual-level monitoring and automated image processing. Cross-discipline collaborations and expanded funding networks that encourage cyclical development and continued technical support are needed to address current limitations and meet the growing need for conservation technologies.

## Introduction

The integration of new technologies for conservation can improve how we monitor and measure changes to species and whole ecosystems^1–3^, which is critical to guide and evaluate management and policy decisions^4^. Technology can provide novel data sources, expanded spatial and temporal coverage, access to real-time information, and rapid processing and analysis for intervention^1,5–7^. For example, the inclusion of real-time transmission and processing of data streams from acoustic devices has advanced remote detection and response to illegal logging^8^. The rapid growth and availability of technologies has been driven largely by adapting existing and consumer-oriented technologies to fit specific conservation needs^9^, including hobby drones for monitoring and response to threats^10,11^, in situ molecular analyses in remote field settings^12^, radar data to forecast bird migrations at continental scales^13^, and the application of blockchain protocols for fisheries supply chain management^14^.

While these options are widely available for commercial application, they may lack features required for ecological conservation purposes such as limited durability and power efficiency, constraints from proprietary silos, or high technical knowledge barriers^15–18^. In other cases, adoption of data-rich and real-time sensors can lead to secondary problems with managing large datasets that often require their own custom approaches and pipelines^3,13,19^. Such constraints are thought to limit the uptake of new tools, but only recently have efforts been made to assess the degree to which they restrict the use of technologies in conservation settings and how to prioritize improvements for future development^15^.

In response to the limitations of off-the-shelf technologies, efforts have grown to actively create novel technologies geared towards conservation^16,20^. Conservation-driven efforts for purpose-built research and monitoring tools include hardware with a lower price compared with private consumer versions^21,22^, development of custom hardware to meet specific needs^23^ and integration of existing platforms for real-time alerts^24^. They may also require collaborations with technologists (defined as experts in technology-related fields including hardware engineering, software development, and machine learning) and companies to produce open-source products for research and management, such as Microsoft’s MegaDetector^25^, Google Earth Engine^26,27^, Vulcan’s EarthRanger^28^, and Wildlife Insights^29^. The bottom-up approach of small scale innovation puts increasing importance on cross-discipline collaborations between end users with first-hand knowledge of real world needs and existing obstacles (i.e., practitioners, researchers, and governments), and technologists, who have the skills to develop and adapt custom technologies^20,30^.

A recent broad survey on the state of the conservation technology field identified that collaboration and information sharing across disciplines and projects was a primary opportunity^15^. For technology-based solutions to have substantial conservation impacts, there is a need for collaborations that effectively identify feature needs, share data, and facilitate iterative development and support^9,15^. To facilitate development of conservation technologies and effectively leverage the support of technologists, we aimed to understand the factors contributing to or inhibiting engagement in the collaborative process of technology development and adoption for field use. We surveyed active conservation practitioners, researchers and conservation-oriented technologists regarding conservation technology development to answer three questions: 1) What are the technical barriers for technological uptake among end-users, and are development priorities focused on alleviating these?; 2) How are conservation technology collaborations structured, and what are the perceived barriers to successful collaborations?; and 3) To guide future development, what upcoming technologies are the conservation community looking for?

## Results

Of the 101 completed survey responses, we categorized respondents into three groups: 53 were conservation practitioners, 42 were academic researchers, and seven were technologists. Familiarity and experience with conservation technologies varied widely among respondents. Most (71%) of respondents reported being extremely or very familiar with technologies, while 26% reported being moderately familiar. Most (96%) respondents also had experience using existing technologies for conservation applications, while fewer had experience in testing new or unproven tools (48%), adaptation or iterative development of existing tools (54%) or design of new tools (34%). Among user groups, more conservation practitioners were engaged in the development of new conservation technologies (71%) compared to academic researchers (45%).

**Figure 1:**
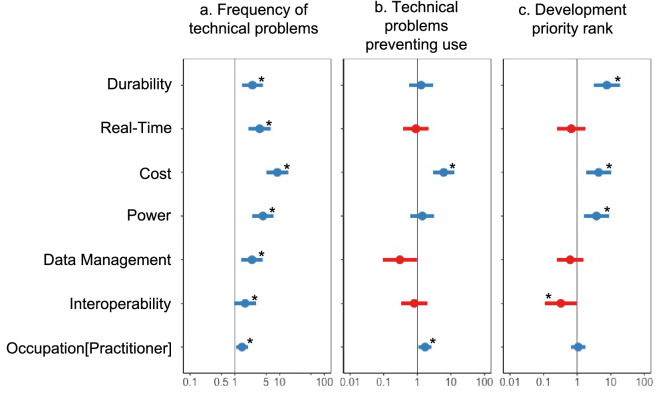
The importance of technology features as barriers to use and development priorities. Coefficient estimates (odds ratios) and 95% confidence intervals are shown for predicted relationships between feature types and (**a**) the frequency of feature-related issues experienced during use, (**b**) the frequency that feature-related issues prevented use of a tool or device, and (**c**) the feature priority in development of new tools and devices. For A and B, blue circles indicate where respondents experienced more problems. For C, blue circles indicate where respondents ranked features with higher priority. Asterixis denote where coefficient estimates and confidence intervals did not overlap 1 and indicate a significant influence.

To address our first question, the technical barriers for using technologies identified by conservation practitioners and academic researchers were similar, highlighting durability (OR = 2.48, 95% CI[1.44 – 4.26]), cost (OR = 8.91, 95% CI[5.07 – 15.65], power efficiency (OR = 4.24, 95% CI[2.45 – 7.35], data management (OR = 2.42, 95% CI[1.39 – 4.22]), and real-time transmission (OR = 3.59, 95% CI[2.03 – 6.35]), (Fig. 1a). However, only cost (OR = 6.10, 95% CI[3.02 – 13.16]) was identified as likely to prevent the use of a technology in the field (Fig. 1b). Development priorities that were highly ranked among practitioners and researchers were aligned with reported technical issues: durability (OR = 7.65, 95% CI[3.09 – 18.97]), cost (OR = 4.34. 95% CI[1.84 – 10.22], and power efficiency (OR = 3.74, 95% CI[1.57 – 8.88]) (Fig. 1c). In the limited responses from technologists, we found feature priorities were focused on cost (7/7 respondents included cost in the top three) and ease of use (4/7). In contrast, durability (2/7 in top three) and power efficiency (1/7) were not highly ranked among technologists (SM Appendix 2 Fig. 1).

For our second question, we recorded 84 unique collaborations ranging from 2 to 15 partners with a median of five partners. Of the collaborations, 93% involved practitioners, 68% involved academic researchers, and 58% involved technologists. Only 29% of collaborations used websites or forum resources (e.g. wildlabs.net), but 75% of these occurred in a collaboration without a tech expert. Technologists were disproportionately involved in the design stage, while practitioners and researchers were mainly involved in the testing and use phase (SM Appendix 2 Fig. 4). Among barriers to collaborations with conservation technology, high cost (53%) was reported most frequently, followed by delayed timelines (41%) and lack of technical support (25%) (SM Appendix 2 Fig. 5). In terms of factors affecting collaboration experience, our model with poor communication between conservationists and technologists (OR = 0.23, 95% CI[0.06–0.96] and high cost (OR = 0.34, 95% CI[0.11–1.02]) was the most parsimonious model in explaining poor collaborations (SM Appendix 2 Table 7).

**Figure 2:**
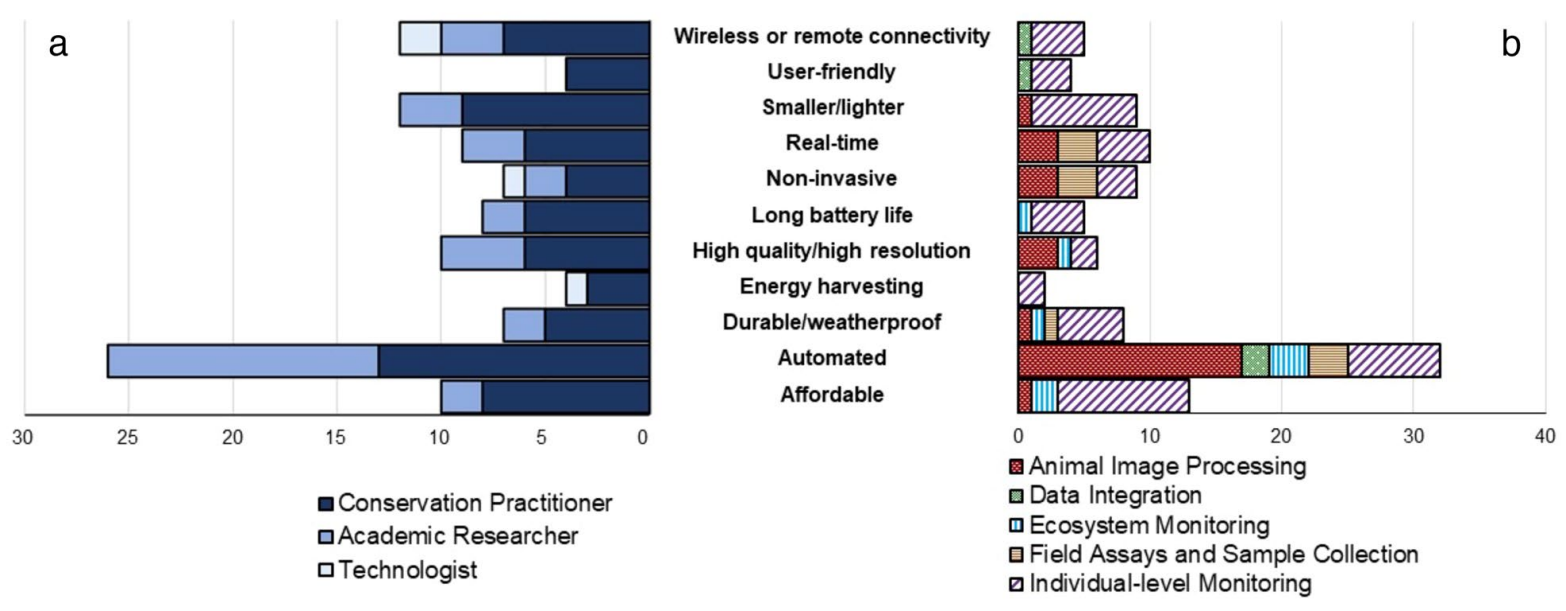
Categories of improvements to existing technology identified by occupation group and application type. The x-axis denotes the counts of respondents. Answers were derived from a theme analysis of the open-ended survey question “Assuming unlimited funding and resources, what technological solution would you want to see developed?”. Theme analysis codebooks can be found in SM Appendix 2 Table 9 and 10.

In response to our third question, we identified several strong themes for desired future technologies. Most of the listed technologies were improvements or extensions to existing tools (e.g. mesh network tracking tags, field-ready genetic analysis kits), while some had specific use cases, such as a device to non-invasively collect and protect hair samples for DNA analysis (SM Appendix 2 Table 9). Automation was mentioned in nearly one third of responses (32/101), with most use cases for automation in reference to animal image processing (53%) and individual-level monitoring (22%) (Fig. 2b). Additionally, researchers were largely focused on automation advancements, while practitioners listed a more diverse set of feature needs (Fig. 2a). For all responses on desired technologies, individual-level monitoring (51%) and animal image processing (28%) were the most-mentioned use cases.

## Discussion

The shift in conservation technology from adaptation of off-the-shelf devices to bottom-up innovation requires a strong collaborative environment and solid understanding of the current and future needs of conservation practitioners and researchers^20^. Our assessment of technical barriers identified frequent issues with multiple feature types, but cost disproportionately prevented the use of technologies in conservation and research settings. While previous studies have touted advanced technologies as a cost-effective pathway to expand the reach and resolution of environmental monitoring^1,10^, our results suggest that the high upfront cost of new technologies puts currently-available tools out of reach for many groups. These costs manifest across device purchase, training and implementation time, maintenance, data storage, and processing. In addition, low cost is often misaligned with other features that respondents identified, such as durable environment-proofing and robust technical support. For example, the popular AudioMoth low-cost acoustic monitoring platform is sold without a protective case for 60 USD, but users can purchase a case for 35 USD^31^. While this extra durability increases the cost by over 50%, the design demonstrates a flexible approach to keep prices low for users who do not require robust environmental protection or can build their own solution.

Our assessment of collaboration structures found that just over half involved a technologist, which may explain the highly reported issues with delayed timelines and lack of technical support. While lack of communication between partners was only reported in 17% of responses, it was the most significant contributor to poor collaborations. This appears to stem from identified issues that end users were under-represented in the development and adaptation stages of the development cycle, and technologists were under-represented in the testing and use phases. While reports of conservation technology failures are not widely reported in the literature, our results align with themes from successful technology applications. For example, the ElephantBook tool^32^ was developed between a team of computer scientists, students, researchers, and conservation managers to aid re-identification of elephants using machine learning. They improved functionality by integrating with existing data platforms and have continued to provide technical support to advance the tool^32^. Similarly, Snapshot Safari^33^ has found success with cyclical development in collaboration with multiple stakeholders and technologists. The project provides a platform for camera trap data processing and has slowly expanded to include more study sites, expanded functionality to allow tagging by citizen scientists, and added machine learning to pre-process images^33^.

Additionally, our limited data from technologists suggest a different set of feature priorities for conservation technologies and highlights the importance of involving end-users from the beginning to ensure that tool specifications meet conservation needs^30^. Potential solutions to this include adopting a ‘lean start-up’ approach used in commercial sectors that seeks to identify end-users, define features prior to development, and iteratively improve on new products^34^. In concert, platforms like Wildlabs.org and events such as technology challenges and hackathons (e.g. conservationxlabs.com) can allow end users and developers to connect around conservation problems and foster cross-discipline collaborations. However, further research on the success and limitations of these avenues could help improve and expand networking options in the future. Further, documenting tools and operating instructions in white papers, publications, or setup and troubleshooting guides (e.g. AuidoMoth Getting Started Guide), could help uptake by end users. In the absence of direct technology support, websites and forums also appeared to be an important source of information among respondents. Conservation technology sites that collate solutions (e.g. Wildtech.mongabay.com) and platforms that facilitate networking and information sharing (e.g. Wildlabs.org) can be a viable solution to alleviate some of the technical knowledge roadblocks to the development and use of technologies in practice.

Our assessment of emerging needs in the conservation technology space identified software-based automation tools as the largest desire. Many respondents referenced the need to handle the increasing size of data streams, suggesting that automation is an important need among the conservation community. This aligns with recent results from a broad survey of the conservation technology field pointing to the need to enhance capacity for large-scale data analyses^15^. Surprisingly, many of the ideas for automation technology already exist in some form, such as automated identification and counting of individual animals in camera trap images^19^. This suggests that scaling new devices and software beyond the original project may prove difficult when most end users lack the technical know-how and infrastructure to adapt it to their specific use case. One example of this scenario is AI-based classification and detection models for camera trap images, where the drift in species assemblages and environments between sites can severely degrade classification performance^25^, and where users require the skills or collaborators to implement models and code from open source repositories^25^. Tools developed by Google’s Wildlife Insights^29^ are now available for researchers to process data for a wide range of species and habitats with a simple user interface. In other cases, automation improvements in one area may lead to secondary problems. For example, real-time tracking data of wildlife using accelerometer sensors can automatically flag immobility due to injury or poaching^3^ but requires in-depth analysis of specificity and sensitivity to improve allocation of management resources (G. Wittemyer pers. comm. 2021).

To reduce existing barriers and meet the emerging needs of conservation professionals through bottom-up innovation, our results point to the importance of an adaptive development process that brings end-users to the table early and keeps developers involved beyond the initial release. In the commercial and industrial sectors, spiral development processes with build-test-feedback-revise iterations are shown to get products to market quicker^35^. Further, companies that focus on the voice of the customer can build better and longer-lasting products^36^. In the conservation sphere however, continual developer support may not always be feasible as pro-bono engineers switch to new projects or grant cycles end^20^. In these cases, establishing a strategy to build financially sustainable products using alternative funding models from the beginning of the project may help sustain the tool beyond the end of the initial funding cycle. Research on financial models for conservation technology are limited^15^, but opportunities include open-source designs that can be community-maintained^37^, social impact enterprises that follow commercial strategies to maximize environmental impact alongside profits^38^, or public–private partnerships that have been used to support technology growth in other underfunded sectors^39^. In concert, the funding network for conservation technologies can encourage best practices of iterative development and continued product support, while reducing cost barriers to scale beyond pilot sites. To achieve the full potential of conservation technologies through small-scale innovation, we must continue to foster collaborations across disciplines, sustain product support, and seek alternative funding models for future tech developments.

## Methods

### Survey

We identified our survey population using groups with a conservation or conservation-technology focus. First, we selected groups for which 1) there was active membership; 2) members were likely to have at least some familiarity with technology for conservation; and 3) it was possible to obtain the number of people that the survey was sent to estimate response rates. We also sought to distribute the survey to groups that would capture practitioners and scientists working in diverse fields and environments. We also identified groups that would have a high percentage of technology experts. Through this process we identified 11 groups: Society for Conservation Biology Working Groups for Freshwater, Conservation Technology, and Animal Behavior in Conservation, Snapshot Safari, Wildlife Insights, Vulcan EarthRanger developers, Smithsonian Institute, San Diego Zoo Wildlife Alliance, Wildlabs, and the AI for Conservation Slack channel. We dropped the Society for Conservation Biology Conservation Technology working group because we received no responses.

The survey instrument (SM Appendix 1) was distributed via email and listserv postings to each group. In the case of the AI for Conservation group, the survey was sent through Slack. Due to privacy requirements, it was not always possible to collect individual email addresses for distribution, so it was possible for a person to receive the survey multiple times if they were a part of different distribution groups. The survey consisted of 24 questions, involving a combination of multiple-choice, Likert-scale^40^, and open-response questions. The survey was designed to answer three overarching research questions: 1) What are the technical barriers for technological uptake among end-users, and are development priorities focused on alleviating these?; 2) How are conservation technology collaborations structured, and what are the perceived barriers to successful collaborations?; and 3) What future technologies are the conservation community looking for?

To better-evaluate these questions, the survey was structured around two ways of interacting with conservation technology: 1) the use of technology tools for conservation and research, and 2) the development (i.e., design, adaptation, and testing) of new tools. Respondents were asked to specify their occupation from a list of 6 options. Due to the limited sample size, occupation was collapsed into three categories: conservation practitioners (front-lines conservationists, non-academia researchers and conservation facilitators), academic researchers (professor/faculty/postdoc and graduate student), and technologists. In conjunction, we defined four distinct roles that respondents could take on: Use of existing and established tools for work, testing of new or unproven tools, adaptation or iterative development of existing tools, and design of new tools. Respondents were allowed to select more than one role. We used skip logic to only show respondents questions relevant to their experience and roles with conservation technology.

We administered the survey online through Qualtrics from 10th July 2020 to 30th October 2020. To access the survey, respondents were required to consent to participate in our study and were assured that their responses would remain completely anonymous. The survey distribution list reached 648 people. Follow-up emails were sent to each group once, approximately one month after the initial email. We received 101 complete responses, for a response rate of 15.6%. Although this rate is relatively low^41^, it is consistent with other online surveys that used email to contact respondents^42,43^. The survey was carried out according to the United States Federal Policy for the Protection of Human Subjects, and all protocols and methods were approved by Colorado State University’s Institutional Review Board before implementation (Protocol No. 20-10050H). Informed consent was obtained from all participants.

### Statistical analysis

Descriptive statistics were reported as percentages. For all models, responses from technologists were withheld and evaluated separately due to low response rates from this occupation group. We conducted all statistical analyses using R version 4.0.3 and the ordinal package^44,45^.

To investigate our first question on technical barriers, we used two questions in the survey. First, to identify the prevalence of issues, we asked respondents to list the frequency at which they encountered different technical limitations. We used an ordinal logistic regression model to evaluate the frequency of occurrence, defined as Never to Always with five categories, in relation to technical limitation and occupation. We categorized seven possible limitations: 1. high or prohibitive cost; 2. lack of durability; 3. poor power efficiency; 4. data access limitations; 5. data management problems; 6. lack of interoperability with other devices or software; or 7. lack of or poor real-time data transmission. Durability was defined to respondents as features that prevented damage to tools (e.g. waterproofing, theft-proofing, etc.). Second, to determine the extent to which technical limitations impacted use of technologies, we asked respondents to indicate whether each limitation had prevented them from using a device or tool in the past. We used a logistic regression model to evaluate the prevention of use in relation to the technical limitation and occupation.

To investigate our question on conservation technology collaborations, we first quantified collaboration structures. For each collaboration, we calculated the percentage of collaborator types involved in each of the four roles (design, adapt, test, and use) of the development process. Collaborator types were collapsed into four categories: practitioners and non-academic researchers, academic researchers, technologists, and website and forum resources. To evaluate barriers, we first summarized the overall frequency of barrier types reported and evaluated the relationship between barriers and collaboration success using ordinal logistic regression models. We used Likert-scale^40^ ratings of collaboration experience, defined as Poor to Excellent with five categories as the response variable (n = 54), and collaboration group size (continuous), type of technology (hardware or software), and collaboration limitations as possible predictors. Collaboration limitations were defined as: 1. high cost; 2. delayed timeline, 3. lack of project management, 4. misunderstanding on deliverables, 5. lack of technical support, 6. poor communication between conservationists and technologists, and 7. lack of partners. To select the most parsimonious model, we first fit a full model that included all covariates. From this full model, we sequentially dropped the least informative covariate (defined by minimum absolute value of b/SE) and refit the model. The higher order model was discarded if eliminating a covariate led to a reduction in AICc, and this approach was carried out until no additional covariate could be eliminated without leading to an increase in AICc^46^.

To investigate our question on emerging needs, we assessed unmet needs using answers derived from a theme analysis of the open-ended survey question “Assuming unlimited funding and resources, what technological solution would you want to see developed?”. SB used NVivo 12 Pro^47^ to inductively (i.e., without predetermined categories) code responses into themes (SM Appendix 2 Table 9, 10). After the initial coding, all authors re-examined, refined, and integrated codes, when necessary, based on our research objectives^48,49^.

## Supplementary Information

Supplementary Information 1.

Supplementary Information 2.

Supplementary Information 3.

## Data Availability

The authors declare that the data supporting the findings of this study are available within the paper and its Supplementary Information files.

## Code availability

The code that supports the ordinal and logistic regression findings presented here is available within the paper and its Supplementary Information files.
