# Supplementary material for: Identifying conservation technology needs, barriers, and opportunities
Source: Sci Rep. 2022 Mar 21;12:4802. doi: 10.1038/s41598-022-08330-w (PMC8938523; doi:10.1038/s41598-022-08330-w)
Supplement: Supplementary file 3 — Supplementary Information 3. [file 41598_2022_8330_MOESM3_ESM.pdf]

Supplementary Materials

Contents

Supplementary Methods

1. Survey instrument
2. Data summaries and model outputs (SM Fig 1-5; SM Table 1-7)

Supplementary Table 8. Codes, definitions, example quotes, and code frequencies from theme analysis of answers to the open-ended survey question "Assuming unlimited funding and resources, what technological solution would you want to see developed?"

Supplementary Table 9. Codes, example quotes, and code frequencies of desired improvements to existing technologies derived from a theme analysis of answers to the open-ended survey question "Assuming unlimited funding and resources, what technological solution would you want to see developed?"

Tech Familiarity

For this survey, technology is defined as any device or software that is used to help conduct research, management, and other conservation-related activities.

Please answer questions based on your last 5 years of experience.

How familiar are you with the use of technology (e.g. camera traps, machine learning, remote sensing) in conservation research and practice?

- ◯ Not familiar at all
- ◯ Slightly familiar
- ◯ Moderately familiar
- ◯ Very familiar
- ◯ Extremely familiar

Horizon Scan

Are there any technological advances, tools, or devices that do not exist, but if developed would allow you to address novel research questions or create new conservation solutions in your field?

- ◯ Yes
- ◯ No

What research or conservation need would the technological solution fill?

- ☐ Species monitoring
- ☐ Ecosystem monitoring
- ☐ Real-time monitoring
- ☐ Automated processing
- ☐ Data integration/visualization
- ☐ Law enforcement
- ☐ Physical asset management
- ☐ [ ] Other

Assuming unlimited funding and resources, what technological solution would you want to see developed?

[ ]

Biodata

Biodata

How would you best describe your position/role?

- ○ Front-lines conservationist
- ○ Conservation facilitator
- ○ Researcher (non-academia)
- ○ Professor/Faculty/Postdoc
- ○ Graduate student
- ○ Technologist
- ○ [ ] Other

Main country(s) of work. List up to three.

Country 1 [ ]

Country 2 [ ]

Country 3 [ ]

What biomes represent your main location of study/work? Select all that apply.
Hold down ctrl (Windows) or cmd (Mac) to select multiple.

Terrestrial
- Tropical Rainforests
- Temperate Forests
- Taiga
- Deserts
- Grasslands
- Savanna
- Tundra
- Urban/Periurban

Wetlands

What best describes your conservation focus? Select all that apply.
Hold down ctrl (Windows) or cmd (Mac) to select multiple.

Research
- Applying/developing research methods
- Biodiversity surveys & mapping species distributions
- Conservation management (implementing or evaluating)
- Conservation planning for species or conservation areas
- Study of species biology
- Wider context of conservation

Threats to Biodiversity
- Climate change
- Disturbance

What best describes your research focus? Select all that apply.
Hold down ctrl (Windows) or cmd (Mac) to select multiple.

Species
- Birds
- Mammals
- Fish
- Reptiles
- Invertebrates
- Amphibians
- Plants
- Bacteria, archaebacteria, fungi, protozoa

Wildlands/Landscapes Conservation

What best desribes the organization that you work for?

- ○ Conservation NGO
- ○ Collaborative research partnership/Institute
- ○ Government agency
- ○ University
- ○ Technology company
- ○ [ ] Other

Years of experience in your field. Field refers to the field most related to your use of conservation technology.

- ○ 0-4 years
- ○ 5-9 years
- ○ 10-14 years
- ○ 15+ years

If you found this survey to be valuable and would like to send it to others, please add contact email addresses here. We will add them to our distribution list.

[ ]

Default Question Block

How have you interacted with conservation/research-centric technology? Select all that apply.

- ☐ Use of technology in the field: use of existing/established tech
- ☐ Adaptation of existing technologies: modifications of existing tech to suit conservation use cases
- ☐ Development of new technologies: creating new tech for conservation use cases
- ☐ Testing of new/adapted technology: trialing of new/unproven tech
- ☐ Participation in technology challenges or outreach
- ☐ I have not interacted directly with conservation technologies

How often do you experience the following technical and performance issues while using technology for conservation research and practice?
For statements that are not applicable to your work, indicate N/A.

	How often do you experience this issue?						Has this issue ever prevented you from using a technology? Click if yes
	N/A	Never	Rarely	Sometimes	Often	Always	Select all that apply
Lack of durability/field-proofing (e.g. theft, waterproofing)	○	○	○	○	○	○	☐
Difficulty of use	○	○	○	○	○	○	☐
Lack of real-time data/connectivity	○	○	○	○	○	○	☐
High cost	○	○	○	○	○	○	☐
Limited power efficiency/battery life	○	○	○	○	○	○	☐
Data access/sharing limitations	○	○	○	○	○	○	☐
Data management challenges	○	○	○	○	○	○	☐
Limited interoperability with existing tools and workflows	○	○	○	○	○	○	☐
Other [ ]	○	○	○	○	○	○	☐

What are the technology tools that you/your team use for collecting data and implementing conservation activities?
To select multiple technologies, hold down ctrl (Windows) or cmd (Mac).

Sensors/Hardware
- Camera traps
- Acoustic sensors
- Tracking tags/collars
- Solar power source equipment
- Cell phone
- Handheld GPS
- UAVs/Drones
- Digital/DSLR cameras
- Seismic sensors

Other

Indicate how you use each technology in conservation settings.

	What application do you use this technology for? Select all that apply.						
	Species Monitoring	Ecosystem Monitoring	Real-time Monitoring	Automated Processing	Data Integration/Visualization	Law Enforcement	Physical Asset Management
» Camera traps	☐	☐	☐	☐	☐	☐	☐
» Acoustic sensors	☐	☐	☐	☐	☐	☐	☐
» Tracking tags/collars	☐	☐	☐	☐	☐	☐	☐
» Solar power source equipment	☐	☐	☐	☐	☐	☐	☐
» Cell phone	☐	☐	☐	☐	☐	☐	☐
» Handheld GPS	☐	☐	☐	☐	☐	☐	☐
» UAVs/Drones	☐	☐	☐	☐	☐	☐	☐
» Digital/DSLR cameras	☐	☐	☐	☐	☐	☐	☐
» Seismic sensors	☐	☐	☐	☐	☐	☐	☐
» 3D Printing	☐	☐	☐	☐	☐	☐	☐
» Low resolution satellite imagery (10+ meter)	☐	☐	☐	☐	☐	☐	☐
» High resolution satellite (sub-10 meter)	☐	☐	☐	☐	☐	☐	☐
» Multispectral	☐	☐	☐	☐	☐	☐	☐
» Thermal	☐	☐	☐	☐	☐	☐	☐
» LiDAR	☐	☐	☐	☐	☐	☐	☐
» Radar	☐	☐	☐	☐	☐	☐	☐
» Machine learning/AI	☐	☐	☐	☐	☐	☐	☐
» Mobile survey platforms (e.g., survey123)	☐	☐	☐	☐	☐	☐	☐
» Imagery processing platforms (e.g. Google Earth Engine)	☐	☐	☐	☐	☐	☐	☐
» Image processing tools (e.g. camera trap labeling)	☐	☐	☐	☐	☐	☐	☐
» Automated image processing tools (e.g. AI-based species ID)	☐	☐	☐	☐	☐	☐	☐
» Audio processing tools	☐	☐	☐	☐	☐	☐	☐
» Automated audio processing tools	☐	☐	☐	☐	☐	☐	☐
» eDNA/Metabarcoding	☐	☐	☐	☐	☐	☐	☐
» Field forensics kits	☐	☐	☐	☐	☐	☐	☐
» Satellite communication	☐	☐	☐	☐	☐	☐	☐

	What application do you use this technology for? Select all that apply.						
	Species Monitoring	Ecosystem Monitoring	Real-time Monitoring	Automated Processing	Data Integration/Visualization	Law Enforcement	Physical Asset Management
» LoRa	☐	☐	☐	☐	☐	☐	☐
» Radio	☐	☐	☐	☐	☐	☐	☐
» Other	☐	☐	☐	☐	☐	☐	☐

Use the matrix to evaluate the frequency of use and performance of each technology.

	How often do you use this technology?	How would you evaluate the overall performance of this technology?
» Camera traps		
» Acoustic sensors		
» Tracking tags/collars		
» Solar power source equipment		
» Cell phone		
» Handheld GPS		
» UAVs/Drones		
» Digital/DSLR cameras		
» Seismic sensors		
» 3D Printing		
» Low resolution satellite imagery (10+ meter)		
» High resolution satellite (sub-10 meter)		
» Multispectral		
» Thermal		
» LiDAR		
» Radar		
» Machine learning/AI		
» Mobile survey platforms (e.g., survey123)		
» Imagery processing platforms (e.g. Google Earth Engine)		
» Image processing tools (e.g. camera trap labeling)		
» Automated image processing tools (e.g. AI-based species ID)		
» Audio processing tools		
» Automated audio processing tools		
» eDNA/Metabarcoding		
» Field forensics kits		
» Satellite communication		
» LoRa		
» Radio		
» Other		

What types of collaborators do you work with to use technologies in conservation research and practice? Select all that apply.
Hold down ctrl (Windows) or cmd (Mac) to select multiple.

Conservation Partners
- Field-based/local conservation group
- International conservation NGO
- Government agency
- Zoo/Sanctuary

Technology/Engineering Partners
- Data scientist
- Hardware engineer
- Generalist/hobbyist
- Program manager

In what context do you work with these groups to implement conservation technologies?

	Select the contexts of each collaboration			
	Use	Testing	Adaptation	Development
» Field-based/local conservation group	☐	☐	☐	☐
» International conservation NGO	☐	☐	☐	☐
» Government agency	☐	☐	☐	☐
» Zoo/Sanctuary	☐	☐	☐	☐
» Data scientist	☐	☐	☐	☐
» Hardware engineer	☐	☐	☐	☐
» Generalist/hobbyist	☐	☐	☐	☐
» Program manager	☐	☐	☐	☐
» Software engineer	☐	☐	☐	☐
» Websites, forums, or online tutorials	☐	☐	☐	☐
» Academia - Conservation, Ecology, Biology	☐	☐	☐	☐
» Academia - Engineering	☐	☐	☐	☐
» Academia - Data science	☐	☐	☐	☐
» Collaborative research partnership/Institute	☐	☐	☐	☐
» No one	☐	☐	☐	☐
» Other	☐	☐	☐	☐

Non-Dev Questions

Did you experience any limitations that prevented you from taking part in conservation technology projects? Select all that apply.

☐ Cost
☐ Timeline
☐ Project management
☐ Lack of understanding on deliverables
☐ Lack of technical support
☐ Misunderstanding between scientist and technologist

- ☐ Lack of partners
- ☐ None
- ☐ [ ] Other

How interested would you be to participate in the development, adaptation, or testing of new conservation technologies in the future?

- ○ Never
- ○ Not very interested
- ○ Neutral
- ○ Somewhat Interested
- ○ Very interested

Dev Questions

The following questions will ask specifically about your experience developing, adapting, and/or testing technologies for conservation applications. For this section, only refer to your most recent experience.

What type of technology did you worked with? Select only one.

Sensors/Hardware
- Camera traps
- Acoustic sensors
- Tracking tags/collars
- Solar power source equipment
- Cell phone
- Handheld GPS
- UAVs/Drones
- Digital/DSLR cameras
- Seismic sensors

How important are the following features for this technology?
Drag and drop to rank. Add non-applicable features to the N/A Group

Items	Ranking Group
Durability	
Ease of Use	
Price	
Power efficiency/battery life	
Data management	**N/A Group**
Interoperability with other tools/systems	
Connectivity/Real-time transmission	
Other [ ]	

What limitations (if any) did you experience during the process? Select all that apply.

- ☐ High cost
- ☐ Delayed timeline
- ☐ Lack of project management
- ☐ Misunderstanding on deliverables
- ☐ Lack of technical support
- ☐ Misunderstanding between scientist and technologist
- ☐ Lack of partners
- ☐ No limitations
- ☐ Other ________

How would you evaluate the experience?

- ○ Very Poor
- ○ Poor
- ○ Acceptable
- ○ Good
- ○ Very Good

Powered by Qualtrics

Supplemental Materials: Data Summaries and Outputs

Nathan Hahn, Sara Bombaci, George Wittemyer

2021-05-07

Figures

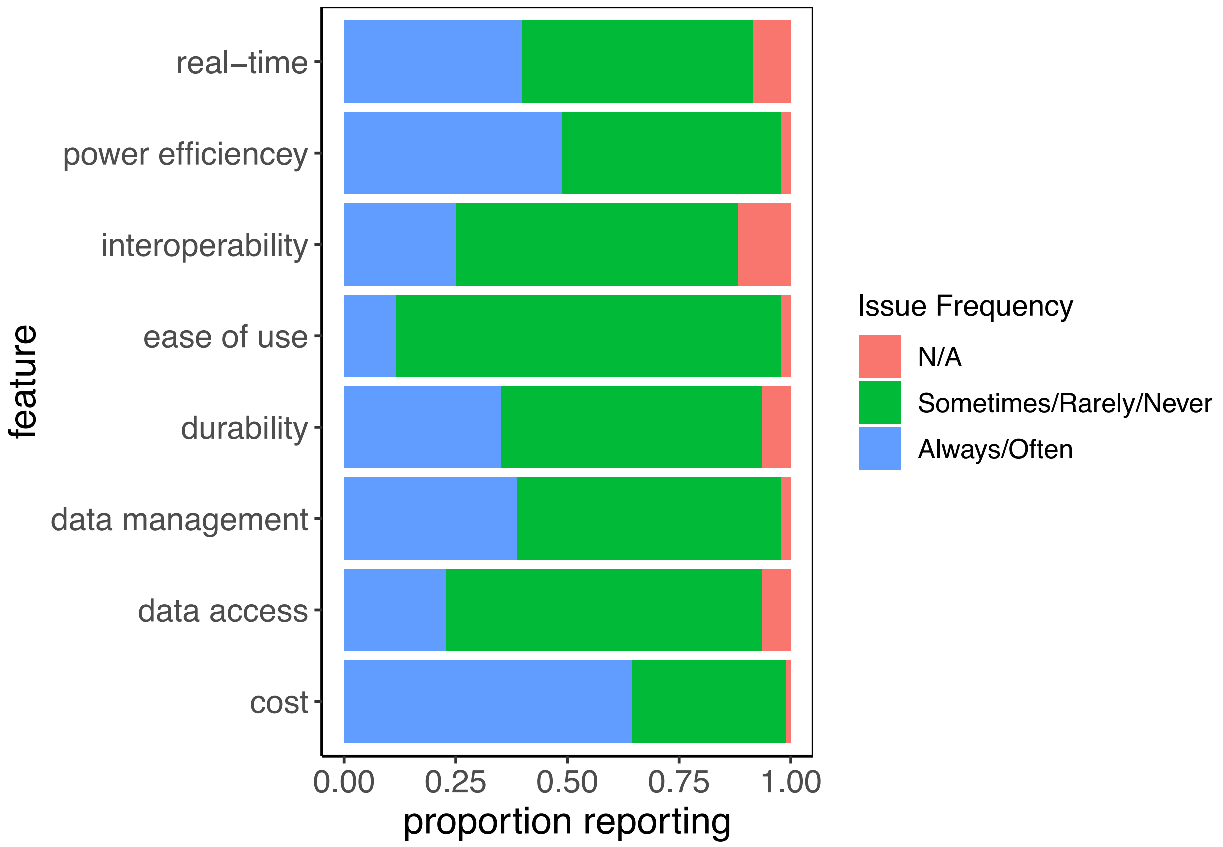

SM Figure 1. Summary of responses to the question "How often do you encounter technical issues with each of the following technology features?". To summarize the data, the frequency of feature issues are condensed into three categories: Always/Often, Sometimes/Rarely/Never, and Not Applicable. Based on these categories, over 50% of respondents experience issues always or often with every feature. Cost is the most frequent issue (77% always/often), but is also the most commonly reported as not applicable (16%). Issues with power efficiency (73% always/often) and durability (69%) were also common.

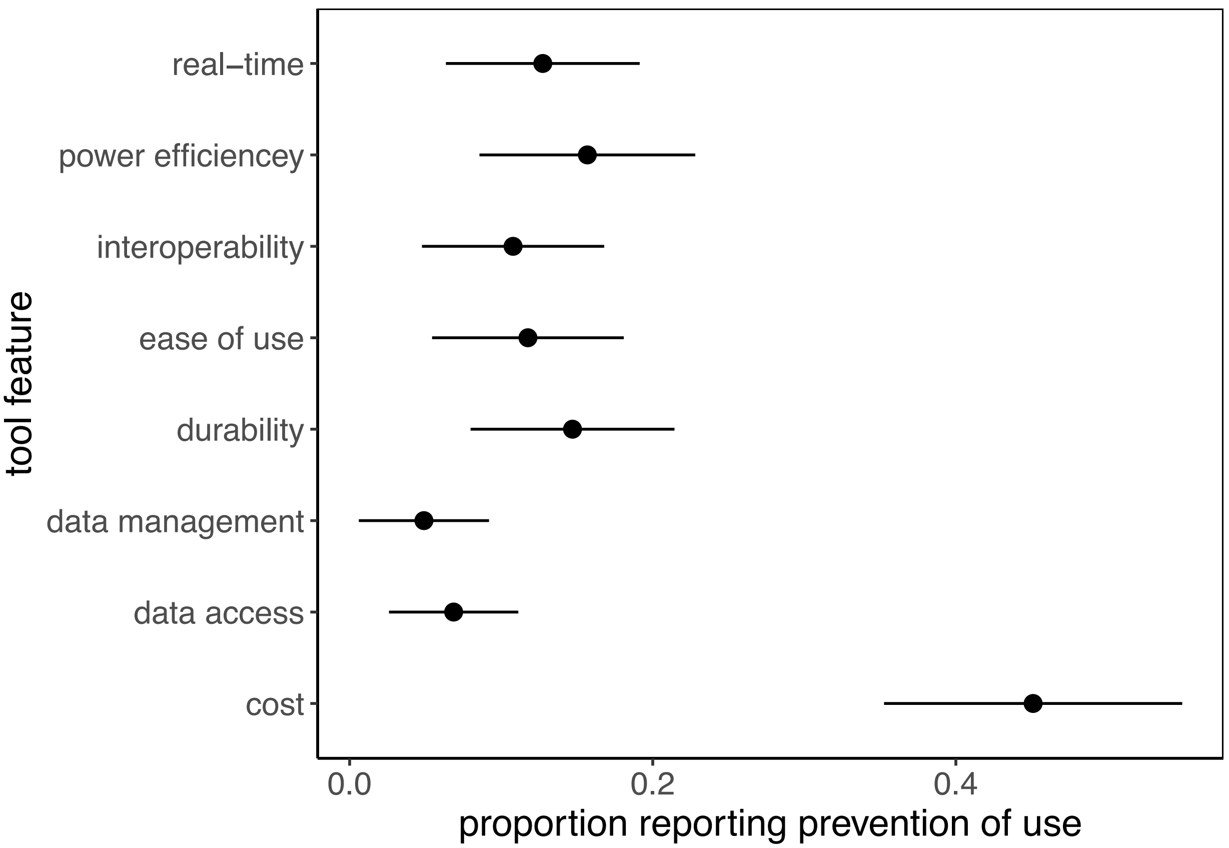

SM Figure 2. Proportion of respondents who were unable to to use a device or tool due to each technical issue (i.e. prevented use). Bars correspond to bootstrapped 95% confidence intervals.

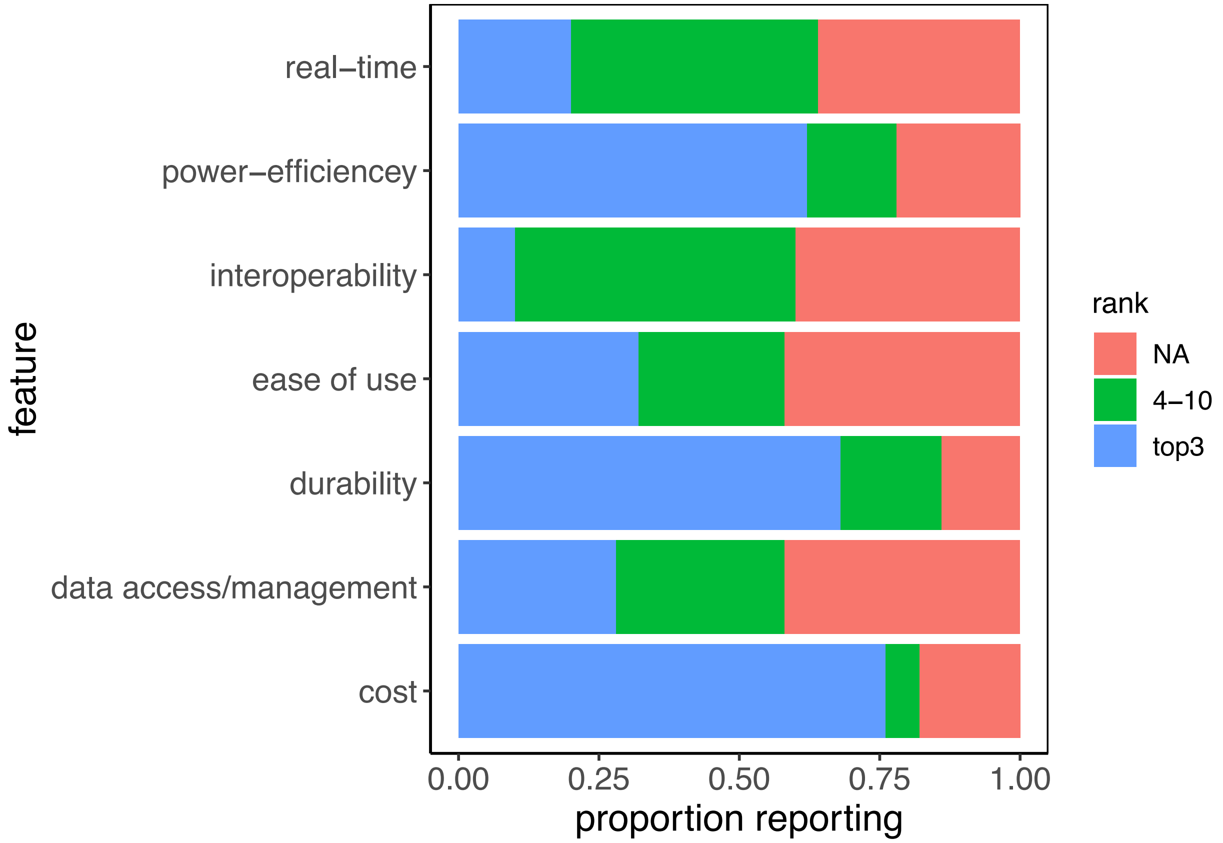

SM Figure 3. Feature priorities for development. To summarize development priorities, respondents who had worked on technology design, adaptation, or testing were asked to rank features by priority for development. Respondents were also able to indicate when a feature was not applicable to their project. Respondents were instructed to base their rankings on a single project and technology. Rankings are collapsed into three categories for plotting: Top 3, 4-10, and Not Applicable.

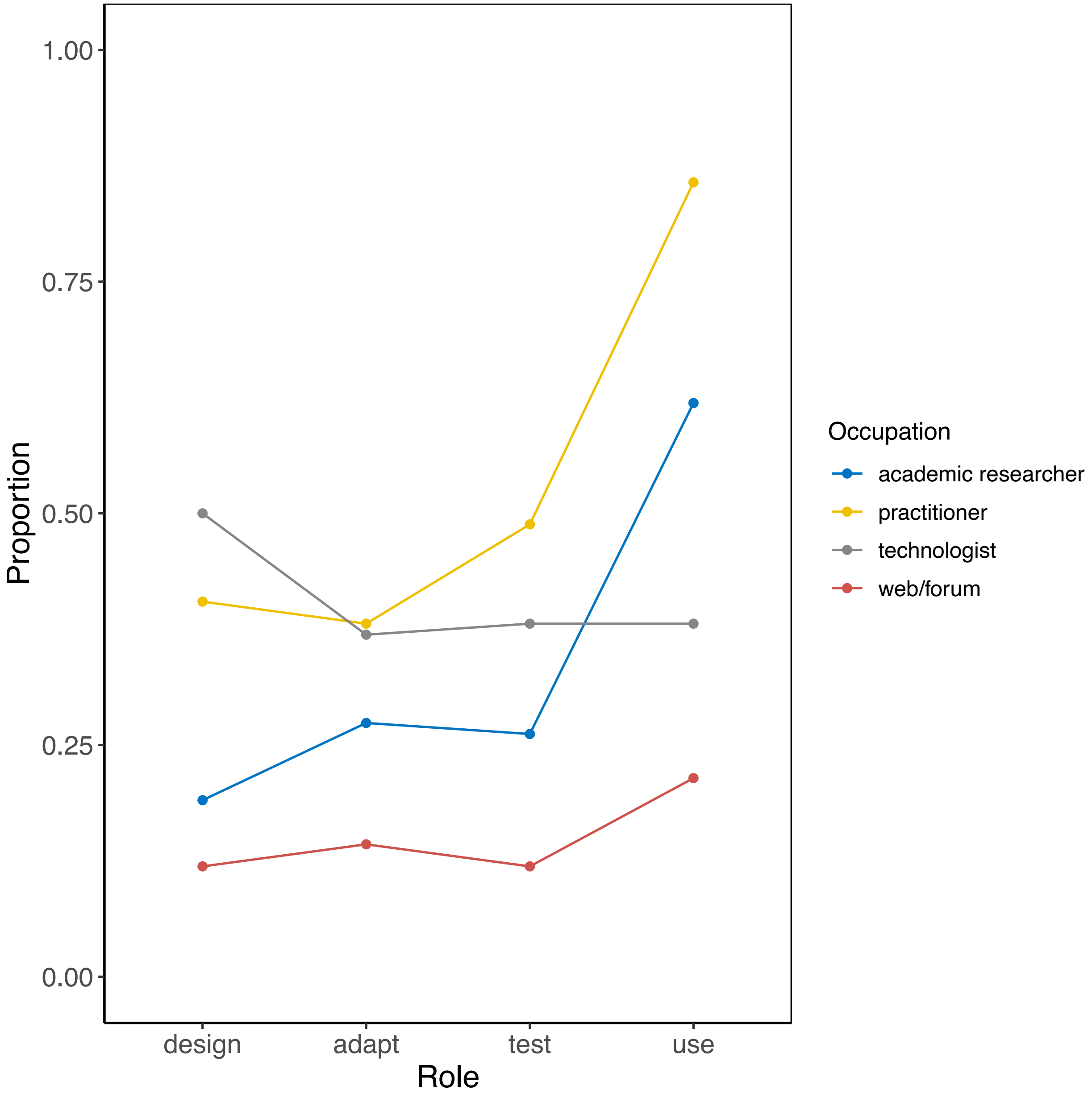

SM Figure 4. Rates of participation in collaborations within each role of the development process. Each colored line corresponds to a different collaborator type. We asked about websites and forums to distinguish when collaborations were turning to online resources and documentation for technical support as opposed to technologists.

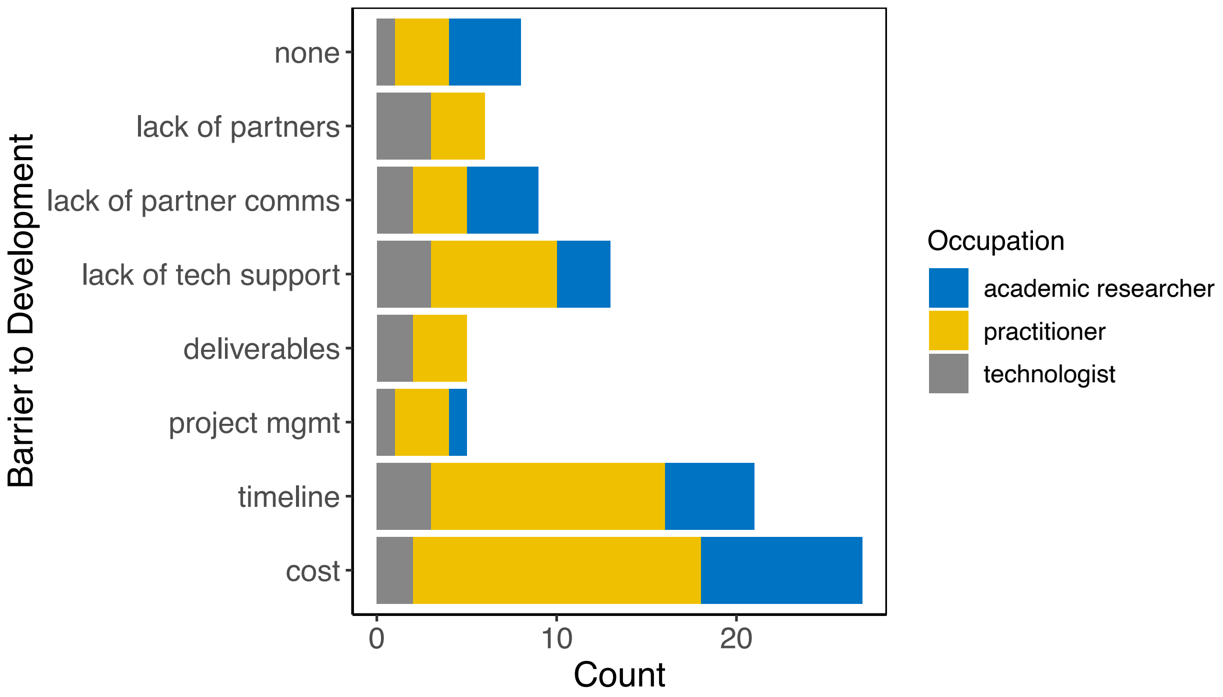

SM Figure 5. Summary of responses to the question 'What barriers do have you experienced in developing conservation technologies?". High cost, delayed time-line, and lack of technical support are the three most commonly-reported barriers.

Tables

SM Table 1. Conservation and research objectives reported in response to the question "What are your main area(s) of conservation focus?"

Conservation Focus	Count	Pct
Study of species biology	48	11.82%
Conservation management (implementing or evaluating)	47	11.58%
Biodiversity surveys & mapping species distributions	44	10.84%
Applying/developing research methods	41	10.10%
Habitat fragmentation/change	35	8.62%
Conservation planning for species or conservation areas	34	8.37%
Disturbance	33	8.13%
Wider context of conservation	28	6.90%
Human-Wildlife Conflict	28	6.90%
Climate change	17	4.19%
Introduced/Invasive species	16	3.94%
Overexploitation/Illegal Take	13	3.20%
Pollution and disease	10	2.46%
Effects of small population size	10	2.46%
Other	2	0.49%

SM Table 2. Summary of technology types in use by the survey respondents. Technologies have been collapsed into 6 overarching categories. Note that respondents could select multiple technologies in use.

Tech Category	Count
Sensors	218
Hardware	174
Software	156
Imagery	84
Comms	35
Biotech	10

SM Table 3. Odds of encountering technical issues with different tool features during use of conservation technologies. Technical issues were assessed using an ordinal regression with logit link. Coefficients are shown as odds ratios with 95% confidence intervals, where confidence intervals not overlapping 1 are considered significant. Positive values indicate a feature was more likely to have technical issues.

Frequency of Technical Issues

	**frequency**	
Predictors	*Odds Ratios*	*CI*
feature [durability]	2.48	1.44 – 4.26
feature [real-time]	3.59	2.03 – 6.35
feature [cost]	8.91	5.07 – 15.65
feature [power]	4.24	2.45 – 7.35
feature [data management]	2.42	1.39 – 4.22
feature [interoperability]	1.70	0.97 – 2.97
occupation [practitioner]	1.45	1.07 – 1.97
Observations	576	
log-Likelihood	-768.274	

SM Table 4. Barriers to implementation of new technologies in conservation settings. In addition to technical barriers that prevent use of technology in the field, we also summarizes responses about implementation barriers when beginning to use new technologies in practice. Note that this was a multiple choice question, so percentages do not sum to 100.

Barrier to Use	Pct	lwr.ci	upr.ci
cost	0.79	0.71	0.88
timeline	0.34	0.24	0.45
lack of tech support	0.30	0.20	0.39
lack of partners	0.22	0.13	0.31
none	0.15	0.07	0.23
project mgmt	0.14	0.07	0.21
deliveriables	0.13	0.06	0.20
lack of partner comms	0.13	0.06	0.19
other	0.06	0.01	0.11

SM Table 5. Odds of a technical issue preventing use of technologies in a conservation setting. Prevention of use was assessing using a regression with logit link. The results are shown with ease of use and academic

researcher as reference variables. 95% confidence intervals are calculated for the odds ratios, where intervals not overlapping 1 are considered significant.

Feature Issues Preventing Use

	prevent	
Predictors	*Odds Ratios*	*CI*
(Intercept)	0.10	0.05 – 0.20
feature [durability]	1.30	0.57 – 3.01
feature [real-time]	0.91	0.37 – 2.18
feature [cost]	6.10	3.02 – 13.16
feature [power]	1.40	0.63 – 3.23
feature [data-mgmt]	0.30	0.08 – 0.91
feature [interoperability]	0.81	0.33 – 1.99
profession_2 [user]	1.70	1.09 – 2.69
Observations	665	
log-Likelihood	-265.974	

SM Table 6. Odds of a high development priority rank for different features when designing conservation technologies. Feature priority rankings were assessed using an ordinal regression with logit link. Coefficients are shown as odds ratios with 95% confidence intervals, where confidence intervals not overlapping 1 are considered significant. Positive values indicate a features was more likely to be ranked highly. We used ranking levels of 1, 2, 3, 4, and 5+, where 5+ contains rankings between 5 and 7. Technologist responses were dropped from the model due to low response rates and evaluated separately.

Feature Priorities for Development

	rank	
Predictors	*Odds Ratios*	*CI*
feature [durability]	7.65	3.09 – 18.97
feature [real-time]	0.66	0.25 – 1.76
feature [cost]	4.34	1.84 – 10.22
feature [power]	3.74	1.57 – 8.88
feature [data-mgmt]	0.62	0.25 – 1.53
feature [interoperability]	0.32	0.11 – 0.97
profession_2 [ac_user]	0.93	0.57 – 1.53
Observations	223	
log-Likelihood	-324.278	

SM Table 7 Factors affecting collaboration ratings of practitioners and academic researchers. Odds ratios and 95% confidence intervals below 1 indicate a negative effect of the predictor on the collaboration rating.

Lack of partner communication had a strong negative effect on collaboration ratings, while high costs had a marginal negative effect.

Collaboration ratings

	collab.rating	
Predictors	*Odds Ratios*	*CI*
cost [1]	0.34	0.11 – 1.02
partner_communication [1]	0.23	0.06 – 0.96
Observations	50	
log-Likelihood	-57.858	

Supplementary Table 8. Codes, definitions, example quotes, and code frequencies from theme analysis of answers to the open-ended survey question "Assuming unlimited funding and resources, what technological solution would you want to see developed?" Responses are coded into different themes representing desired applications.

Codes	Definition	Example Quote	Code Frequency
Data Integration	Tools for processing large volumes of data, analytical tools for integrating multiple data sources, integrating drones with GPS collars, and integrating collar data with modelling	"software facilitating better data integration; specifically computational power and tools to integrate and process large volumes of data easily; user friendly tools for developing deep-learning and machine learning analysis"	6
Ecosystem Monitoring	Automated image processing to detect land use change for conservation management, to quantify river discharge, or collect polarized light measurements	"Suite of tools for protected area management, streamlined. Allow countries / PAs to get a higher resolution picture of the ecosystem conditions and ecosystem service valuation of their regions"	9
Animal Image Processing	Automated species or individual animal identification from sensors ; distance to animal measurements, measures of weight/mass, bait detection	"automated image processing for detecting animals in satellite and drone imagery"; "automated species recognition & individual ID on camera traps"; "ability to estimate density from camera traps for non-uniquely marked individuals"; "a device to record the mass of individual animals that pass in front of camera traps"; "AI that can recognize bait in camera traps"; "measurement of physiological variables in camera traps"	29
Individual-level Monitoring	Improvements to animal tracking devices and adaptations to measure individual animal behaviour and physiology	"Miniaturized animal tracking technologies with extended battery life;" "camera system on animals which links to automated behaviour software"	24
Field Assays and Sample Collection	sex assays from swabs, hormone analysis of samples, field-ready genetic analysis, dietary information from terrestrial	"ID animals instantly as male/female from swabs/test strips"; "rapid hormone analysis"; "Near real time dietary	9

vertebrate faecal isotope data, tool to collect hair samples and protect from moisture/weather; tag attachment/retrieval options that do not require capture and immobilization; tool to explore burrows; thermal nest cameras; stereo cameras for 3D wildlife images	information from faecal isotope data"; "mechanism to collect hair samples for DNA analysis that would also protect those samples from moisture"; "Automated on-site parasite identification and quantification portable device"

Supplementary Table 9. Codes, example quotes, and code frequencies of desired improvements to existing technologies derived from a theme analysis of answers to the open-ended survey question "Assuming unlimited funding and resources, what technological solution would you want to see developed?"

**Improvements of Existing Technology**	**Example Quote**	**Code Frequency**
Affordable	"Soundscape monitoring; tracking animals in a feasible manner, ie., accessible for researchers with less access to funds"	10
Automated	"Automated identification of individual animals based on spot/stripe patterns"; "Automated integration of collaring data with habitat suitability modelling"; "automated drone mapping of all river/stream habitats with LiDAR/sonar"	26
Durable, weatherproof, waterproof	"Better waterproofing for most of the technology we use"; "Sand-proof DSLR cameras"; "Collars with better battery life & collar straps"	8
Self-powered/energy harvesting	"Remotely deployable solar GPS tags"; "Energy harvesting smart cameras with long range connectivity"	4
High quality or high resolution	"good camera trap which will be small as GoPro8 and will produce high quality 10 MP pictures with high iso and will last half year in the field"; "Cameras that triggered faster and with greater sensitivity"; "More sensitive acoustic sensors (enhanced range of detection)"	10
Long battery life	"Drones that can monitor a conservation area for hours. This means a long lasting btterylife"; "Increased battery life for underwater cameras"	8
Non-invasive	"less invasive tracking devices"; "non-invasive genetic analysis in the field"; "Noninvasive aerial insect monitoring"	7
Real-time	"ID animals instantly as male/female from swabs/test strips"; "Mesh network telemetry tags and receivers(real-time, lowest power/kilobyte): tag/receiver proximity measurements, tags as mobile environmental sensors, data storage and forwarding from tags outside the receiver network)"	9
Smaller or Lighter	"miniaturized tracking tag/collars"; "smaller, high-resolution all-weather camera traps"	13
User-friendly	"user friendly tools for developing deep-learning and machine learning analysis"; "easy to implement VHF triangulation systems"	4

Wireless or remote connectivity	“Better data connectivity from the field” ;“improved cell phone, smartphone, and network access for communities living around wildlife conservancies; particularly in developing / low-income regions”; “B.A.T.M.A.N. (Better Approach to Mobile Ad-hoc Networking) for LoRa”	12
